# Post COVID 19 multisystem inflammatory syndrome in an older adult

**DOI:** 10.1080/0886022X.2021.1895839

**Published:** 2021-03-15

**Authors:** Alexander Parpas, Michael Yudd, Albert W. Dreisbach, Jennine Michaud

**Affiliations:** VA New Jersey Healthcare System, East Orange, New Jersey

Dear Editor,

The novel coronavirus disease (COVID 19) caused by SARS-CoV-2 has caused widespread illness and death across the world and we continue to identify new sequelae of this disease. We present a case of a post COVID 19 multisystem inflammatory syndrome in an older adult with debilitating neurological and gastrointestinal manifestations and acute kidney injury (AKI) requiring hemodialysis. There have been over 1000 cases of post COVID 19 multisystem inflammatory syndrome in children (MIS-C) described, but none in older adults. The exact mechanism of MIS-C is unknown but is believed to be due to a delayed cytokine storm related to the initial infection. Given the vast number of cases of COVID 19 worldwide, we should be conscious of the possibility of a post COVID 19 multisystem inflammatory syndrome in adults as we work toward effectively treating this virus.

We present a 67-year-old male with a past medical history of cirrhosis and hypertension, diagnosed with COVID 19 pneumonia 68 days prior. During the initial admission for COVID 19 pneumonia, he required oxygen *via* high flow nasal cannula and was treated with doxycycline, ceftriaxone, vitamin C, hydroxychloroquine, zinc, therapeutic enoxaparin, thiamin, dexamethasone, and convalescent plasma. Inflammatory markers were initially high but improved prior to discharge. He returned to our institution 3 weeks after discharge with complaints of generalized weakness, anorexia, and weight loss. He also had nausea, dyspnea on exertion, lower extremity swelling and cognitive difficulties, but denied fevers. He was admitted with tachycardia, leukocytosis, AKI and severe hyponatremia. Initial blood pressure 151/98 mmHg, heart rate 115 beats per minute, respiratory rate 20 breaths per minute, temperature 98.1 °F, and oxygen saturation 96% on room air. On physical examination, he was tachycardic with a regular rhythm, clear lungs with edema in all extremities and generalized muscle weakness. Labs revealed sodium of 109mEq/L, WBC of 35.9 K/cmm, creatinine of 1.1 mg/dL (Table 1). Chest x-ray revealed bibasilar infiltrates, CT showed atelectasis. Urinalysis showed protein 100 mg/dL, moderate blood, 10 WBC/hpf, 5 RBC/hpf, and muddy brown casts. SARS-CoV-2 PCR was negative, but antibody testing was positive. Echocardiogram showed grade 1 diastolic dysfunction, elevated pulmonary pressures, and normal left ventricular ejection fraction. Hyponatremia corrected appropriately with normal saline. Leukocytosis persisted despite treatment with broad-spectrum antibiotics, and all cultures were negative. Due to a high d-dimer and clinical suspicion for pulmonary embolus, therapeutic unfractionated heparin and thrombotic evaluation were initiated. Ventilation/perfusion scan was inconclusive for pulmonary embolism; lower extremity duplex was negative for deep vein thrombosis.

Renal function deteriorated and hemodialysis was initiated. Percutaneous renal biopsy showed moderate to marked acute tubular necrosis (ATN), no significant fibrosis, inflammation, immune complexes, or viral inclusions. Given that the patient was never hypotensive and AKI developed prior to the hospitalization, ATN was attributed to cytokine surge. He required 5 treatments of hemodialysis before renal function improved. Due to persistent symptoms and elevated inflammatory markers, dexamethasone 6 mg daily was started due to concern for a post COVID 19 multisystem inflammatory syndrome. After 4 days, he improved and was discharged on a prednisone taper. Follow up showed rapid improvement in symptoms and significant reduction in inflammatory markers after 15 days of steroid initiation. His appetite normalized, he gained 10 pounds, and was able to walk 150 feet. BNP and inflammatory markers decreased significantly correlating with improvement in symptoms.

## Discussion

COVID 19 caused by SARS-CoV-2 causes a wide spectrum of illness ranging from an asymptomatic carrier state to multiple organ failure and death. After exposure, the onset of symptoms typically occurs within 14 days, median incubation period of 4–5 days. The average recovery period ranges from 2–6 weeks depending on severity of illness [1]. The morbidity associated with COVID 19 is related to complications from the initial infection, which include respiratory failure, renal failure, thrombotic events, inflammatory complications, and secondary infections. Recently, there have been reports of a multisystem inflammatory state in children, MIS-C, primarily in older children and adolescents, occurring after the initial infection resolved. Many children were asymptomatic carriers with antibodies to SARS-CoV-2 and a negative PCR. This post COVID 19 multisystem inflammatory phenomenon has not been well described in adults.

The MIS-C definition includes 6 criteria: serious illness leading to hospitalization, age less than 21 years of age, fever >38 °C or subjective fever lasting at least 24 h, laboratory evidence of inflammation, multisystem organ involvement and laboratory evidence of SARS-CoV-2 infection (positive SARS CoV-2 PCR or antibody testing). Active surveillance yielded 186 patients in 26 states in the United States. Median age was 8.3 years of age, male predominance, 70% had a positive PCR or antibody test, 73% were previously healthy, and 92% had at least 4 biomarkers indicating inflammation [2].

Over 2000 cases of MIS-C worldwide with onset 2–4 weeks after initial infection, incidence of 2 in 100,000 people under age 21, and most with antibodies against SARS- CoV-2. Clinical features included elevated troponin and B-type naturietic peptide (BNP), elevations in inflammatory markers such as C-reactive protein, ferritin, and d-dimer. Additional findings included anemia, lymphopenia, and hypoalbuminemia [3]. The initial cytokine storm caused by SARS-CoV-2 is a major cause of acute respiratory distress syndrome (ARDS) and multiple organ failure. There were several inflammatory cytokines (IL-1B, MCP-1, IL-4 and IL-10) found in patients with COVID 19 able to activate either Th1 or Th2 cells. They are believed to play a key role in the development of ARDS and damage to extrapulmonary organs such as the kidneys. A high viral titer and alteration of the cytokine/chemokine response can cause a cytokine storm and subsequent tissue damage [4]. A late cytokine storm has been described in children with MIS-C. The exact mechanism is unknown, but several hypotheses have been proposed. One theory suggested an aberrant cellular or humoral adaptive immune response given the late onset of symptoms. Antibodies may enhance the severity of COVID 19 by triggering inflammation or organ damage or be part of a post-infectious event [3,5]. Another theory considered genetic variation in genes regulating the T- and B-cell response to the clearance of immune complexes [3]. Additionally, the initial infection may trigger an autoimmune or autoinflammatory condition through molecular mimicry, or predispose one to environmental insults [6]. Finally, as the viral load increases and/or genetic factors slow antiviral responses, the viral replication can delay the interferon response and the cytokine storm can result before adaptive responses clear the virus, resulting in MIS-C [5].

We believe post COVID 19 multisystem inflammatory syndrome in adults will be more pronounced as cases of COVID 19 continue to rise. Diagnostic criteria should include older patients so that they are recognized quickly and treated appropriately. Our patient had 4 of the 6 criteria included in the MIS-C criteria but was not in the specified age range and did not have a fever. He had the clinical features including elevated inflammatory markers, evidence of cardiac involvement with an elevated BNP, and hematological abnormalities. After two weeks of treatment with steroids he had neurologic, gastrointestinal and renal improvement in symptoms and significant reduction in inflammatory markers.

**Table 1.** Post-COVID-19 multisystem inflammatory syndrome.

Labs	Admission #1	Discharge	Admission #2	Steroid initiation	Day 15 after steroid initiation
White blood cells (4.5−10 K/cmm)	5.9	8.2	35.9	12.3	12
Neutrophils % (54−62)	63.6	59.6	91	64.7	74.9
Lymphocytes % (24−44)	19.5	27.5	1	26.8	21.1
Hemoglobin (14−17.5 gm/dL)	16.6	11.5	10.1	9.3	9.9
Platelets (150−450 K/cmm)	325	346	651	354	318
Serum sodium (136−145 mEq/L)	136	140	109	142	143
Serum creatinine (0.7−1.3 mg/dL)	1.1	0.6	1.1	2.4	1.1
Albumin (3.5−5.7 g/dL)	3	2.9	1.9	2.6	3.9
D-dimer (<500 ng/mL)	3626	244	2276	12,109	1089
Lactate dehydrogenase (140−271 U/L)	531	213	294		216
BNP (0−100 pg/mL)	19		449		161
CRP (0–3 mg/L)	244.9	16.4	141.1	143.4	6.3
ESR (0–15 mm/hr)	45		100	128	77
Ferritin (23.9–336.2 ng/mL)	1094.6	790	1280.9		825
Procalcitonin (0–0.5 ng/mL)	0.62		1.66		
Spot urine protein to creatinine ratio			1.76		0.38
